# Effects of Barbell Position and Squat Depth on Trunk and Lower-Extremity Muscle Activation During the Back Squat in Healthy Adult Men

**DOI:** 10.3390/medicina62071339

**Published:** 2026-07-11

**Authors:** Ju-Hyung Park, Shi Lei, Hwi-Young Cho, Sung-Hyeon Kim

**Affiliations:** 1 Department of Health Science, Gachon University Graduate School, Incheon 21936, Republic of Korea; parkpt91@gmail.com (J.-H.P.); shilei1997@gachon.ac.kr (S.L.); 2 Department of Physical Therapy, Gachon University, Incheon 21936, Republic of Korea

**Keywords:** electromyography, lower extremity, resistance training, back squat, muscle activation

## Abstract

*Background and Objectives*: The back squat is widely used in resistance training, performance enhancement, and rehabilitation, and barbell position and squat depth are key modifiable variables that may influence neuromuscular demand. This study investigated the effects of barbell position and squat depth on trunk and lower-extremity muscle activation during the back squat. *Materials and Methods*: Twenty-two adult men with at least 2 years of back-squat experience performed high-bar and low-bar back squats to knee-flexion angles of 50°, 90°, and 130° using a standardized external load corresponding to 80% of each participant’s habitual one-repetition maximum (1RM). Conditions were presented in randomized order. Surface electromyography was used to record the rectus femoris, vastus medialis, vastus lateralis, gluteus maximus, semitendinosus, biceps femoris, rectus abdominis, and erector spinae. Electromyographic activity was analyzed separately for eccentric and concentric phases and normalized to maximal voluntary isometric contraction (%MVIC). *Results*: Increasing squat depth most consistently increased quadriceps and erector spinae activation, indicating greater knee extensor and trunk extensor demand at deeper positions. Rectus abdominis activation showed a depth-related effect during the concentric phase, with only isolated pairwise differences during the eccentric phase, whereas gluteus maximus activation showed limited modulation. Barbell position produced selective phase- and depth-dependent responses: the low-bar squat showed greater hamstring and erector spinae activation in specific comparisons, whereas the high-bar squat showed greater knee-extensor activation at selected depths, most clearly in the rectus femoris. *Conclusions*: Barbell position and squat depth modify relative neuromuscular demand during the back squat and may be used to adjust the emphasis on knee extensor, trunk stabilizer, and posterior-chain demands rather than to target a single muscle group in isolation.

## 1. Introduction

With increasing recognition of physical activity importance, participation in health-oriented leisure-time exercise has increased. Resistance training benefits cardiovascular, musculoskeletal, metabolic, and psychological domains [1,2], and is recommended at least twice per week [3,4]. Accordingly, resistance training is an essential component for improving physical fitness and maintaining health. Among resistance exercises, the squat enhances muscle coordination, functional performance, and balance, and is used in rehabilitation and injury-prevention programs [5,6].

Rehabilitation paradigms have shifted from machine-based exercises emphasizing isolated strength gains to functional movement-based interventions that simulate activities of daily living [7]. The squat reflects everyday movements such as sit-to-stand and promotes trunk stabilization and combined hip and knee extension through multi-joint coordination [5]. This functional approach is supported by evidence showing improvements in strength recovery, joint stability, and neuromuscular control with load progression [8,9]. In addition, modifiable parameters including stance width, foot rotation, trunk and tibial inclination, and squat depth influence joint moments and muscular demands, allowing knee-dominant or hip-dominant strategies to be selected according to clinical needs [5]. Accordingly, squats are applied after hip or knee surgery, and progressive loading improves strength and functional recovery [10,11].

Squat biomechanics and muscle activation patterns vary with execution. Among modifiable variables, barbell position and squat depth influence joint moments, center-of-mass displacement, muscle recruitment patterns, and ground reaction forces [12,13]. Barbell back squats are classified as high-bar (HBBS) or low-bar (LBBS) based on barbell placement. In HBBS, the barbell is positioned across the upper trapezius, allowing a more upright trunk and greater knee extension moments, whereas in LBBS it is placed lower across the scapular region, increasing forward trunk lean and hip moments [12,14]. These differences influence trunk inclination, joint moment distribution, and trunk stability demands [15,16].

Squat depth is a key variable determining muscle activation, joint loading, and exercise intensity [5]. As squat depth increases, hip and knee flexion increases, and muscle activation and joint loading increase [17,18]. Recent studies have also reported that squat depth influences lower-extremity muscle development and activation [19,20]. Taken together, barbell position and squat depth interactively influence lower-limb muscle recruitment and trunk stability during squat performance.

Most previous studies have examined single factors, with limited attention to combined effects on muscle activation [21,22]. In addition, inconsistent depth definitions and limited quantitative reporting of knee flexion angles have reduced between-study comparability [17]. Moreover, prior research has focused primarily on lower-extremity musculature, with limited consideration of trunk involvement [23,24].

Based on previous evidence and biomechanical considerations, we formulated the hypothesis that barbell position and squat depth would produce significant differences in trunk and lower-extremity muscle activation during the back squat. We further hypothesized that the differences associated with barbell position would vary according to squat depth. Therefore, the present study aimed to quantitatively examine the effects of barbell position and squat depth on trunk and lower-extremity muscle activation.

## 2. Materials and Methods

### 2.1. Participants

Participants were recruited by advertisement at an M center in Incheon, Republic of Korea, from 1 December 2025, describing the study purpose, procedures, eligibility criteria, and participation. Inclusion criteria, based on previous research [25], were healthy adult men aged 20–35 years with at least 2 years of resistance training experience and regular squat training at approximately 80% of one-repetition maximum (1RM) for at least 6 months. Exclusion criteria, adapted from Lorenzetti et al. [13], included musculoskeletal pain or surgery of the spine or lower extremities, neurological disorders, leg-length discrepancy of ≥10 mm, skin conditions at electrode sites, or hypersensitivity to electrode materials. Participants completed a prescreening questionnaire and eligibility screening, and 22 participants met all criteria. All participants provided written informed consent. The study was approved by the Institutional Review Board of Gachon University (Approval No. 1044396-202510-HR-223-01; approval date: 21 November 2025), registered with the Clinical Research Information Service (Registration No. KCT0011241), and conducted in accordance with the Declaration of Helsinki.

An a priori sample size calculation was performed using G*Power (version 3.1.9.7; Heinrich Heine University Düsseldorf, Düsseldorf, Germany) with one group and six measurements. Assuming a conventional moderate effect size (f = 0.25) [26], α = 0.05, power = 0.80, a correlation among repeated measures of 0.50, and a nonsphericity correction of 1.00 (df = 5), the required sample size was 19; allowing for 10% dropout, 22 participants were recruited. Because the original calculation was not interaction-specific, a sensitivity analysis based on the final sample of 22 showed a minimum detectable barbell position × squat depth interaction effect of f = 0.28 (df = 2).

### 2.2. Experimental Design and Procedure

**Figure 1:**
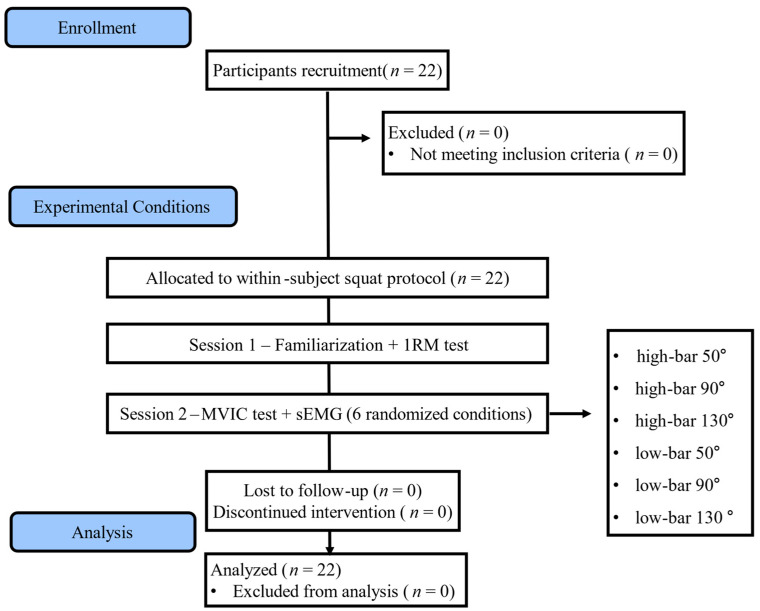
Flow chart of the study. 1RM, one-repetition maximum; MVIC, maximum voluntary isometric contraction; sEMG, surface electromyography; *n*, number of participants.

The study employed a within-subject repeated-measures design; participants performed high-bar and low-bar back squats at knee-flexion angles of 50°, 90°, and 130°, yielding six conditions (2 barbell positions × 3 depths). Condition order was randomized using an online randomization tool (Research Randomizer) (Figure 1).

Experimental procedures were conducted over two sessions separated by 3 days. In the first session, participants completed familiarization and 1RM testing. In the second session, MVIC testing and sEMG measurements during the six squat conditions were performed. Before 1RM testing, participants completed a 5 min cycle ergometer warm-up. The 1RM was determined using a progressive loading procedure adapted from previous protocols [27,28]. Loads were increased from submaximal levels based on each participant’s habitual 1RM estimate, with 2.5–5 kg increments and 3 min of rest between attempts. Testing was performed at 90° of knee flexion using each participant’s habitual back-squat technique, including self-selected stance width and barbell position. A successful attempt required one unaided repetition to 90° of knee flexion followed by a return to the upright position with proper technique. No fixed maximum number of attempts was imposed; one retry at the same load was permitted after an unsuccessful attempt, and failure of the retry terminated testing. The highest successfully completed load was recorded as the 1RM. A standardized external load corresponding to 80% of this habitual 1RM was applied to all six conditions to provide a high external load while allowing consistent technique across repeated conditions; because condition-specific 1RM was not assessed, the load was not interpreted as representing equivalent relative intensity across barbell positions or squat depths.

sEMG was recorded from the dominant limb, defined as the preferred kicking leg [29], and participants were instructed to avoid vigorous exercise, alcohol, and insufficient sleep before testing.

For each condition, participants performed three repetitions using the standardized external load corresponding to 80% of the measured habitual 1RM at knee-flexion angles of 50°, 90°, and 130°, with all conditions performed in randomized order [30]. Movement tempo was controlled using a metronome set at 60 beats per minute, with 3 s for the eccentric phase and 3 s for the concentric phase [31,32]. A 2 min rest interval was provided between conditions, with an additional 5 min rest after maximum voluntary isometric contraction (MVIC) trials. All testing was conducted under consistent environmental conditions.

### 2.3. Squat Task and Load Conditions

All squat conditions were performed using a standardized technique. Participants stood with their feet shoulder-width apart, with the toes oriented forward or slightly externally rotated to about 10°. Participants were instructed to maintain foot contact with the ground, with weight distributed toward the midfoot-heel region. The thorax was kept elevated with the scapulae retracted and depressed to establish trunk bracing, while the lumbar spine was maintained in a neutral or slightly lordotic position. The gaze was directed forward or slightly upward, with head and neck in neutral alignment. The descent was initiated with a hip hinge, allowing forward tibial translation per individual anthropometrics while avoiding heel lift. Participants were instructed to align the knees with the direction of the toes and to maintain approximately parallel alignment between the trunk and tibia. The descent–ascent tempo was standardized at 3 s–3 s using a metronome set at 60 beats per minute [9,33]. Participants initiated the descent on the first beat, reached the target depth on the fourth beat, and returned to the upright position on the seventh beat.

### 2.4. Bar Position Conditions

**Figure 2:**
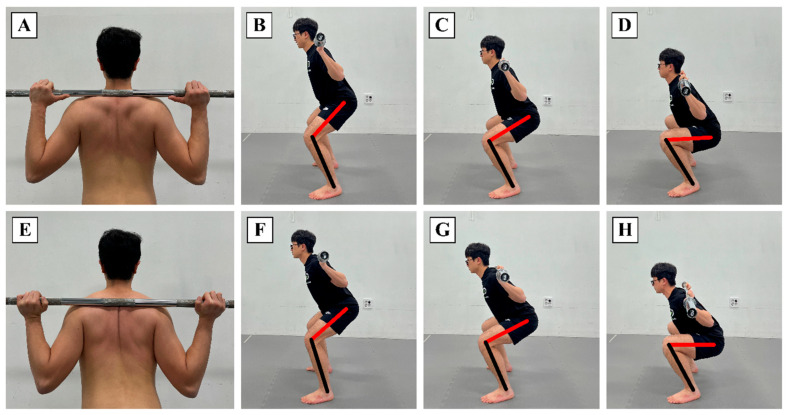
Bar position and squat depth conditions. (**A**) High-bar back squat, (**B**) high-bar 50°, (**C**) high-bar 90°, (**D**) high-bar 130°, (**E**) low-bar back squat, (**F**) low-bar 50°, (**G**) low-bar 90°, (**H**) low-bar 130°. The black and red lines represent the longitudinal axes of the lower leg and thigh, connecting the lateral femoral epicondyle to the lateral malleolus and greater trochanter, respectively.

Participants performed two variations of the barbell back squat. In the high-bar back squat (HBBS), the barbell was positioned across the upper trapezius. In the low-bar back squat (LBBS), the barbell was placed immediately inferior to the spine of the scapula, resting on the posterior deltoid [12] (Figure 2). Bar height and grip width were individually adjusted during familiarization and kept constant for all experimental trials. Participants met the general back-squat experience criterion, but technique-specific prior experience with HBBS and LBBS was not formally recorded. Familiarization was repeated as needed, without a fixed number of sets or repetitions. Practice continued until participants could reproduce the assigned bar position, target depth, and prescribed tempo without major technique deviation.

### 2.5. Squat Depth Conditions

Squat depth was defined on the basis of knee joint flexion angle and set to three conditions: 50° (partial squat), 90° (half squat), and 130° (full squat) [18,30] (Figure 2). Target knee-flexion angles were determined using a goniometer before testing. During familiarization, visual feedback and investigator instruction were provided to help participants reproduce each target depth. Squat trials were recorded with the camera (Samsung Electronics Co., Ltd., Suwon, Republic of Korea) positioned perpendicular to the sagittal plane on the dominant side. The knee-flexion angle was measured on the end-of-descent frame by the investigator using the greater trochanter, lateral femoral epicondyle, and lateral malleolus as anatomical landmarks. Trials deviating by more than 10% from the target knee-flexion angle or failing to reproduce the instructed squat technique were repeated. All depth conditions were presented in a randomized order.

### 2.6. Surface Electromyography Measurement

Surface EMG activity was recorded using an EMG acquisition system (MP160, BIOPAC Systems Inc., Goleta, CA, USA) and AcqKnowledge software (version 6.0.1, BIOPAC Systems Inc., Goleta, CA, USA). Signals were sampled at 1000 Hz and band-pass filtered using a fourth-order Butterworth filter with effective cut-off frequencies of 20 and 450 Hz. Disposable Ag/AgCl electrodes were applied in a bipolar configuration after skin shaving and cleansing with 70% alcohol. Electrode placement followed SENIAM recommendations [34] and previous sEMG procedures used during loaded squat tasks [35] for RF, VM, VL, GM, ST, BF, RA, and ES (Table 1). The reference electrode was placed over the proximal anterior tibial shaft. To normalize EMG amplitude, MVICs were obtained for each muscle using standardized manual muscle testing positions based on Kendall’s procedures [36]. Three 5 s MVIC trials were performed for each muscle, with 1 min of rest between trials. The mean RMS value from the middle 3 s of each MVIC trial was calculated, and the highest value across the three trials was used as the reference MVIC. After completion of all MVIC measurements, a 5 min rest period was provided before the squat trials. Trial onset was defined as the first point at which the rectified and smoothed EMG signal from any monitored muscle exceeded its standing baseline mean by 2 SD. This point was set as 0 s, and the signal was segmented according to the metronome-paced timing into 0–3 s eccentric and 3–6 s concentric phases, with 3 s corresponding to the prescribed maximum-depth transition. Mean EMG activity for each phase was normalized to %MVIC. Raw EMG signals were full-wave rectified and smoothed using a 50 ms moving RMS window, and the mean of three repetitions was analyzed.

### 2.7. Statistical Analysis

Statistical analyses were performed using SPSS Statistics version 25.0 (IBM Corp., Armonk, NY, USA). Descriptive characteristics are presented as mean ± standard deviation. The normality of EMG variables was examined using the Shapiro–Wilk test. For each muscle and movement phase, a two-way repeated-measures analysis of variance was performed with barbell position (high-bar back squat and low-bar back squat) and squat depth (50°, 90°, and 130°) as within-subject factors. The main effects of barbell position and squat depth and the barbell position × squat depth interaction were examined. Mauchly’s test was used to assess sphericity, and the Greenhouse–Geisser correction was applied when the assumption of sphericity was violated. Bonferroni-adjusted pairwise comparisons were performed separately for each muscle and movement phase. The three bar-position comparisons at 50°, 90°, and 130° constituted one comparison family, whereas the three depth comparisons (50° vs. 90°, 50° vs. 130°, and 90° vs. 130°) within HBBS and LBBS constituted separate comparison families. Effect sizes for the repeated-measures ANOVA were reported as partial eta squared. The significance level was set at *p* < 0.05.

## 3. Results

### 3.1. General Characteristics

Twenty-two participants met the inclusion criteria and completed all squat conditions. No protocol deviations or dropouts occurred. No adverse events were reported. All data were collected and analyzed. The general characteristics of the participants are presented in Table 2.

### 3.2. Muscle Activity in the Eccentric Phase

The two-way repeated-measures ANOVA results and Bonferroni-adjusted pairwise comparisons for the eccentric phase are presented in Table 3, Table 4 and Table 5. A significant barbell position × squat depth interaction was observed only for RF (*p* = 0.004). Significant main effects of barbell position were found for RF, VM, BF, ST, GM, and ES (*p* < 0.05), whereas RA and VL did not show significant bar-position effects. Significant main effects of squat depth were observed for RF, VM, VL, ST, and ES (*p* < 0.05), whereas RA, BF, and GM did not show significant depth effects.

Bonferroni-adjusted bar-position comparisons at each squat depth are shown in Table 4, and Bonferroni-adjusted depth comparisons within each barbell position are shown in Table 5. For RF, significant bar-position differences were observed at 90° and 130°, but not at 50°. For ST and ES, significant bar-position differences were observed at all three squat depths. For VM, a significant bar-position difference was observed only at 90°. An isolated bar-position difference for RA was observed at 50° (adjusted *p* = 0.025). For GM, the overall bar-position main effect was significant, but none of the depth-specific pairwise comparisons reached significance after Bonferroni adjustment. Pairwise depth differences were observed for RA, RF, VM, VL, ST, and ES; for RA, only the 50° versus 130° comparison within HBBS was significant (adjusted *p* = 0.012). The specific pairwise *p*-values are presented in Table 5.

### 3.3. Muscle Activity in the Concentric Phase

The two-way repeated-measures ANOVA results and Bonferroni-adjusted pairwise comparisons for the concentric phase are presented in Table 6, Table 7 and Table 8. Significant barbell position × squat depth interactions were observed for RF (*p* = 0.023) and ST (*p* = 0.002). Significant main effects of barbell position were found for RA, RF, VM, VL, BF, ST, and ES (*p* < 0.05), whereas GM did not show a significant bar-position effect. Significant main effects of squat depth were observed for RA, RF, VM, VL, BF, and ES (*p* < 0.05), whereas ST and GM did not show significant depth effects.

Bonferroni-adjusted bar-position comparisons at each squat depth are shown in Table 7, and Bonferroni-adjusted depth comparisons within each barbell position are shown in Table 8. For RF and VL, significant bar-position differences were observed at 90° only. For BF, significant bar-position differences were observed at 50° and 90°, but not at 130°. For ST, significant bar-position differences were observed at 50° and 90°, but not at 130°. For ES, a significant bar-position difference was observed at 50° only. RA showed a borderline bar-position comparison at 130° (*p* = 0.050). The depth comparisons showed significant depth-related differences for RA, RF, VM, VL, BF, and ES, with the specific pairwise *p*-values presented in Table 8.

## 4. Discussion

The present study examined how barbell position and squat depth influence trunk and lower-extremity muscle activation during the back squat. The main finding was not that a single squat condition uniformly increased activation across all muscles, but rather that barbell position and squat depth redistributed neuromuscular demand in a phase- and muscle-specific manner. Increasing squat depth produced the most consistent increases in quadriceps and erector spinae activation, whereas barbell position selectively altered hamstring, trunk extensor, and quadriceps responses depending on movement phase and squat depth. These findings support the view that squat technique variables should be interpreted as modifiers of relative neuromuscular demand rather than as isolated strategies for activating a single muscle group [12,17,23,37,38,39].

Squat depth appeared to operate primarily as a demand-scaling variable for the knee extensor and trunk extensor systems. As knee flexion increased from 50° to 130°, RF, VM, and VL activation increased in both movement phases, indicating that deeper squat positions required greater active control of knee flexion during descent and greater knee-extension output during ascent [17,20,23]. The parallel increase in ES activation suggests that the demand of deeper squatting was not limited to the lower extremity; rather, deeper positions likely increased the need to maintain trunk orientation and resist the tendency toward trunk flexion under the barbell [12,16,37]. This combined quadriceps–erector spinae response indicates that squat depth modifies both lower-extremity and trunk neuromuscular demands, which is consistent with previous evidence that changes in squat depth and loading alter relative muscular effort, joint loading, and trunk-lower-extremity biomechanics [16,17,20,23]. However, the depth effect should not be generalized to all muscles, because the abdominal, hamstring, and gluteal responses were phase- or muscle-specific rather than uniformly depth-dependent.

The abdominal and hamstring responses provide further evidence that the effects of squat depth and barbell position were not uniform across muscle groups. RA activation showed no significant eccentric-phase main effects of squat depth or barbell position, despite isolated pairwise differences between bar positions at 50° and between 50° and 130° within HBBS, whereas a significant depth effect was observed during the concentric phase. This pattern suggests that RA activity may be more closely related to the stabilization demand of rising from deeper squat positions than to a consistent bar-position-specific abdominal response. In loaded squatting, abdominal activity is better interpreted as part of trunk stiffness and lumbopelvic control rather than as evidence that a specific squat variation selectively targets the rectus abdominis [12,40,41]. The hamstring response was also muscle-specific. BF showed a clearer depth-related response during the concentric phase, whereas ST demonstrated a significant barbell position × squat depth interaction without a significant concentric depth main effect. This distinction suggests that hamstring contribution during the back squat should not be treated as a single uniform response, because the biarticular role of the hamstrings allows their contribution to vary as hip and knee angles change across squat depths and barbell positions [12,31,37,38].

GM activation showed limited modulation across squat depths and barbell positions, which qualifies the interpretation of posterior-chain demand. Although GM is a primary hip extensor, GM activation during loaded exercise is influenced by exercise mechanics, external load, movement velocity, stabilization requirements, and task configuration [42]. In the present study, increasing squat depth did not produce a clear depth-dependent GM response, despite consistent increases in quadriceps and ES activation. Although an eccentric-phase bar-position main effect was observed for GM, this did not translate into significant depth-specific bar-position differences, suggesting that the posterior-chain bias associated with LBBS was expressed more clearly through hamstring and trunk extensor activation than through GM activation. This interpretation is consistent with evidence that GM activation varies across lower-body exercises and may be greater in hip-dominant tasks, such as the barbell hip thrust, than during the back squat [42,43]. Therefore, limited GM modulation should be interpreted not as an absence of hip-extensor demand, but as an indication that back-squat variations may distribute extensor demand across multiple synergistic muscles rather than selectively increasing gluteus maximus activation [35,42,43,44].

Barbell position further modified eccentric-phase activation, but this effect was expressed selectively across muscles. The clearest LBBS-related response was observed in ST and ES, both of which showed greater activation in LBBS than in HBBS across all squat depths. This pattern is consistent with the lower bar position increasing forward trunk inclination and shifting part of the eccentric control demand toward the trunk extensors and hamstrings [12,37,38]. In contrast, the HBBS condition showed a knee-extensor-biased response mainly through RF, which was greater in HBBS at 90° and 130°. However, this quadriceps response was not uniform, because VM differed only at 90° and VL did not show significant depth-specific bar-position differences. Therefore, during the eccentric phase, LBBS appeared to produce a more consistent increase in ST and ES activation, whereas the HBBS-related knee extensor response was most evident in RF rather than across the entire quadriceps group [12,15,17,37].

During the concentric phase, the effect of barbell position was more depth-specific than during the eccentric phase. BF and ST activation were greater in LBBS at 50° and 90°, but not at 130°, suggesting that the lower bar position may increase hamstring contribution primarily at partial-to-half squat depths during ascent. This pattern may reflect the greater forward trunk inclination and hip-dominant loading strategy associated with LBBS, which can increase the need for hip extensor contribution and knee joint stabilization during the upward phase [12,37,38]. In contrast, RF and VL activation were greater in HBBS only at 90°, whereas VM did not show significant depth-specific bar-position differences. This indicates that the HBBS-related knee extensor response during ascent was most apparent at an intermediate squat depth and was not expressed uniformly across all quadriceps muscles [12,15,17]. Taken together, the concentric-phase findings suggest that barbell position modifies muscle activation most clearly at selected joint configurations, rather than producing a consistent HBBS or LBBS effect across the full depth range [12,37,38].

From a practical perspective, the present findings suggest that barbell position and squat depth should be used to adjust the relative emphasis of the back squat rather than to target a single muscle in isolation. HBBS may be useful when the goal is to bias the exercise toward knee extensor demand, particularly when greater RF involvement is desired, but this should not be generalized to a uniform increase across all quadriceps muscles [12,15,17,37]. LBBS may be considered when greater hamstring and trunk extensor contribution is desired, especially in individuals who can tolerate increased forward trunk inclination and maintain appropriate trunk control [12,21,37,38]. The RA response should be interpreted within this trunk-control context rather than as evidence that either bar position functions as a specific abdominal-strengthening strategy [39,40]. In addition, the limited modulation of GM suggests that when preferential gluteal activation is a primary goal, back-squat variations may need to be complemented with hip-dominant or unilateral exercises rather than relying solely on changes in barbell position or squat depth [35,42,43,44]. Therefore, barbell position and squat depth may be combined within a periodized exercise prescription to adjust the balance among knee extensor, trunk stabilizer, and posterior-chain demands according to training or rehabilitation goals [5,39,45].

Several limitations should be considered. The relatively small sample size and inclusion of healthy, squat-trained adult men limit generalizability. Because condition-specific 1RM was not assessed, the standardized external load derived from habitual 1RM may have represented different relative intensities across barbell positions and squat depths. Technique-specific prior experience with HBBS and LBBS and familiarization volume were not formally quantified; therefore, residual differences in technical proficiency across conditions cannot be excluded. In addition, muscle activity was assessed only using surface EMG from the dominant limb, and bilateral symmetry of muscle activation was not evaluated. The absence of concurrent kinetic measurements also constrained mechanical interpretation. Although a 2 min rest interval was provided between squat conditions and condition order was randomized, objective fatigue indicators were not measured; therefore, residual fatigue across repeated conditions cannot be completely excluded. The use of a standardized stance and tempo may not reflect real-world variability, and the analysis was limited to acute neuromuscular responses.

## 5. Conclusions

The present study demonstrated that barbell position and squat depth modulate trunk and lower-extremity muscle activation during the back squat in a phase- and muscle-specific manner. Increasing squat depth produced the most consistent increases in quadriceps and ES activation, indicating greater knee extensor and trunk extensor demand at deeper positions. Barbell position selectively redistributed muscle activation: HBBS was associated with a knee-extensor-biased response, most evident in RF, whereas LBBS was associated with greater hamstring and trunk extensor activation under specific phase and depth conditions. GM activation showed limited modulation across conditions, suggesting that posterior-chain demand should not be inferred solely from gluteus maximus activity. These findings indicate that barbell position and squat depth can be used as practical prescription variables to adjust relative knee extensor, trunk stabilizer, and posterior-chain demands during back-squat exercise.

## Figures and Tables

**Table 1:** Attachment of electromyography electrodes.

Muscle	Attachment Region
VM	On the line between the ASIS and the medial ligament, 3/4 way
VL	On the line from the ASIS to the lateral side of the patella, 2/3 way
RF	On the line from the ASIS to the superior of the patella, 1/2 way
BF	On the line between the ischial tuberosity and the lateral epicondyle of the tibia, 1/2 way
ST	On the line between the ischial tuberosity and the medial epicondyle of the tibia, 1/2 way
RA	2 cm lateral to midline at the level of the umbilicus
ES	Interspace at the level of L1 spinous process about 2–3 cm from the midline
GM	On the line between the sacral vertebrae and the greater trochanter, 1/2 way

VM, vastus medialis; VL, vastus lateralis; RF, rectus femoris; BF, biceps femoris; ST, semitendinosus; RA, rectus abdominis; ES, erector spinae; GM, gluteus maximus.

**Table 2:** Characteristics of participants (*n* = 22).

Variables	Mean ± SD
Age (year)	29.00 ± 3.06
Height (cm)	174.21 ± 4.20
Weight (kg)	73.58 ± 9.14
BMI (kg/m^2^)	24.22 ± 2.56
Training experience (year)	2.95 ± 0.97
1RM (kg)	101.76 ± 16.29
Standardized experimental load (kg; 80% of habitual 1RM)	81.41 ± 13.03

BMI, body mass index; 1RM, one-repetition maximum.

**Table 3:** Muscle activity and two-way repeated-measures ANOVA results during the eccentric phase.

Muscle	Bar Position	50°	90°	130°	Bar Effect	Depth Effect	Bar × Depth
RA	HBBS	3.64 ± 1.54	3.97 ± 1.69	4.76 ± 2.56	F = 2.66 *p* = 0.121 ηp^2^ = 0.129	F = 2.93 *p* = 0.092 ^†^ ηp^2^ = 0.140	F = 0.97 *p* = 0.358 ^†^ ηp^2^ = 0.051
	LBBS	4.44 ± 2.19	6.03 ± 8.23	7.63 ± 9.95			
RF	HBBS	12.51 ± 8.14	25.61 ± 17.08	35.65 ± 16.59	F = 8.69 ***p* = 0.009** ηp^2^ = 0.325	F = 52.39 ***p* < 0.001** ^†^ ηp^2^ = 0.744	F = 6.43 ***p* = 0.004** ηp^2^ = 0.263
	LBBS	11.42 ± 6.44	18.66 ± 10.69	29.24 ± 12.18			
VM	HBBS	34.82 ± 16.23	48.78 ± 22.07	57.38 ± 28.29	F = 6.53 ***p* = 0.020** ηp^2^ = 0.266	F = 32.25 ***p* < 0.001** ^†^ ηp^2^ = 0.642	F = 1.99 *p* = 0.152 ηp^2^ = 0.099
	LBBS	34.59 ± 16.34	43.47 ± 19.21	51.52 ± 21.99			
VL	HBBS	40.15 ± 23.03	52.56 ± 22.75	61.17 ± 27.63	F = 4.15 *p* = 0.057 ηp^2^ = 0.187	F = 35.46 ***p* < 0.001** ^†^ ηp^2^ = 0.663	F = 2.04 *p* = 0.145 ηp^2^ = 0.102
	LBBS	40.23 ± 21.37	47.42 ± 24.15	57.47 ± 26.61			
BF	HBBS	19.27 ± 9.66	20.19 ± 13.28	21.69 ± 13.58	F = 6.24 ***p* = 0.022** ηp^2^ = 0.257	F = 0.84 *p* = 0.397 ^†^ ηp^2^ = 0.044	F = 0.54 *p* = 0.524 ^†^ ηp^2^ = 0.029
	LBBS	22.36 ± 11.94	22.20 ± 11.61	23.30 ± 13.46			
ST	HBBS	19.49 ± 12.44	19.26 ± 13.95	17.76 ± 10.71	F = 25.77 ***p* < 0.001** ηp^2^ = 0.589	F = 6.95 ***p* = 0.003** ηp^2^ = 0.279	F = 2.91 *p* = 0.087 ^†^ ηp^2^ = 0.139
	LBBS	24.31 ± 13.49	23.42 ± 14.82	20.54 ± 11.48			
GM	HBBS	21.04 ± 17.34	19.94 ± 13.89	19.91 ± 13.41	F = 6.18 ***p* = 0.023** ηp^2^ = 0.256	F = 0.14 *p* = 0.778 ^†^ ηp^2^ = 0.008	F = 0.54 *p* = 0.585 ηp^2^ = 0.029
	LBBS	21.80 ± 16.58	21.73 ± 14.82	21.79 ± 14.23			
ES	HBBS	33.33 ± 19.59	41.93 ± 20.90	49.61 ± 21.61	F = 20.80 ***p* < 0.001** ηp^2^ = 0.536	F = 31.45 ***p* < 0.001** ^†^ ηp^2^ = 0.636	F = 2.78 *p* = 0.076 ηp^2^ = 0.134
	LBBS	53.47 ± 27.43	58.95 ± 27.43	62.13 ± 25.34			

Note. Values are presented as mean ± SD. RA, rectus abdominis; RF, rectus femoris; VM, vastus medialis; VL, vastus lateralis; BF, biceps femoris; ST, semitendinosus; GM, gluteus maximus; ES, erector spinae; HBBS, high-bar back squat; LBBS, low-bar back squat; F, F-statistic; *p*, *p*-value; ηp^2^, partial eta squared; ^†^, Greenhouse–Geisser-corrected *p*-value. Bold *p*-values indicate statistically significant main or interaction effects.

**Table 4:** Bonferroni-adjusted *p*-values for bar-position comparisons at each squat depth during the eccentric phase.

Muscle	50°	90°	130°
RA	**0.025**	0.752	0.390
RF	1.000	**0.006**	**0.042**
VM	1.000	**0.020**	0.187
VL	1.000	0.080	0.275
BF	0.201	0.305	0.206
ST	**<0.001**	**0.002**	**0.014**
GM	1.000	0.147	0.098
ES	**0.002**	**0.002**	**0.002**

Note. Values are Bonferroni-adjusted *p*-values. RA, rectus abdominis; RF, rectus femoris; VM, vastus medialis; VL, vastus lateralis; BF, biceps femoris; ST, semitendinosus; GM, gluteus maximus; ES, erector spinae. Bold values indicate statistical significance after Bonferroni adjustment.

**Table 5:** Bonferroni-adjusted *p*-values for depth comparisons within each bar position during the eccentric phase.

Muscle	HBBS 50–90°	HBBS 50–130°	HBBS 90–130°	LBBS 50–90°	LBBS 50–130°	LBBS 90–130°
RA	0.115	**0.012**	0.204	1.000	0.415	0.341
RF	**<0.001**	**<0.001**	**<0.001**	**0.005**	**<0.001**	**<0.001**
VM	**<0.001**	**<0.001**	**0.002**	**<0.001**	**<0.001**	**0.001**
VL	**<0.001**	**<0.001**	**0.001**	**0.002**	**<0.001**	**0.005**
BF	1.000	0.936	0.502	1.000	1.000	0.877
ST	1.000	0.096	0.402	0.259	**<0.001**	**0.035**
GM	1.000	1.000	1.000	1.000	1.000	1.000
ES	**<0.001**	**<0.001**	**0.003**	**0.011**	**0.006**	0.291

Note. Values are Bonferroni-adjusted *p*-values. RA, rectus abdominis; RF, rectus femoris; VM, vastus medialis; VL, vastus lateralis; BF, biceps femoris; ST, semitendinosus; GM, gluteus maximus; ES, erector spinae; HBBS, high-bar back squat; LBBS, low-bar back squat. Bold values indicate statistical significance after Bonferroni adjustment.

**Table 6:** Muscle activity and two-way repeated-measures ANOVA results during the concentric phase.

Muscle	Bar Position	50°	90°	130°	Bar Effect	Depth Effect	Bar × Depth
RA	HBBS	3.98 ± 1.60	4.44 ± 1.71	7.76 ± 6.11	F = 6.75 ***p* = 0.018** ηp^2^ = 0.273	F = 9.37 ***p* = 0.006** ηp^2^ = 0.342	F = 2.75 *p* = 0.092 ηp^2^ = 0.133
	LBBS	5.20 ± 3.13	5.64 ± 4.04	10.58 ± 9.81			
RF	HBBS	22.16 ± 16.03	50.97 ± 29.32	63.85 ± 28.15	F = 10.82 ***p* = 0.004** ηp^2^ = 0.375	F = 48.04 ***p* < 0.001** ηp^2^ = 0.727	F = 4.58 ***p* = 0.023** ηp^2^ = 0.203
	LBBS	18.79 ± 9.80	35.90 ± 17.58	58.87 ± 26.01			
VM	HBBS	55.86 ± 28.34	80.06 ± 49.79	92.76 ± 58.37	F = 10.66 ***p* = 0.004** ηp^2^ = 0.372	F = 28.58 ***p* < 0.001** ηp^2^ = 0.614	F = 1.44 *p* = 0.249 ηp^2^ = 0.074
	LBBS	54.10 ± 33.34	68.09 ± 34.71	86.30 ± 48.51			
VL	HBBS	61.63 ± 33.10	84.44 ± 42.36	94.89 ± 47.79	F = 16.36 ***p* < 0.001** ηp^2^ = 0.476	F = 31.65 ***p* < 0.001** ηp^2^ = 0.637	F = 3.00 *p* = 0.080 ηp^2^ = 0.143
	LBBS	59.09 ± 33.43	71.79 ± 41.40	90.72 ± 46.31			
BF	HBBS	22.36 ± 12.05	26.85 ± 15.96	32.73 ± 15.96	F = 18.50 ***p* < 0.001** ηp^2^ = 0.507	F = 17.74 ***p* < 0.001** ηp^2^ = 0.496	F = 1.26 *p* = 0.288 ηp^2^ = 0.065
	LBBS	26.96 ± 12.69	29.96 ± 15.63	35.06 ± 16.13			
ST	HBBS	20.17 ± 14.85	21.13 ± 16.20	22.38 ± 11.77	F = 10.85 ***p* = 0.004** ηp^2^ = 0.376	F = 0.30 *p* = 0.647 ηp^2^ = 0.016	F = 9.26 ***p* = 0.002** ηp^2^ = 0.340
	LBBS	25.68 ± 16.54	26.07 ± 17.03	23.23 ± 13.74			
GM	HBBS	24.19 ± 18.80	26.37 ± 17.00	28.75 ± 16.65	F = 1.10 *p* = 0.309 ηp^2^ = 0.057	F = 1.48 *p* = 0.242 ηp^2^ = 0.076	F = 2.59 *p* = 0.097 ηp^2^ = 0.126
	LBBS	26.38 ± 19.92	28.78 ± 19.81	27.96 ± 17.64			
ES	HBBS	41.60 ± 30.01	55.18 ± 27.76	73.53 ± 34.15	F = 9.28 ***p* = 0.007** ηp^2^ = 0.340	F = 50.40 ***p* < 0.001** ηp^2^ = 0.737	F = 2.51 *p* = 0.101 ηp^2^ = 0.123
	LBBS	57.07 ± 30.63	66.19 ± 36.57	77.91 ± 34.47			

Note. Values are presented as mean ± SD. RA, rectus abdominis; RF, rectus femoris; VM, vastus medialis; VL, vastus lateralis; BF, biceps femoris; ST, semitendinosus; GM, gluteus maximus; ES, erector spinae; HBBS, high-bar back squat; LBBS, low-bar back squat; F, F-statistic; *p*, *p*-value; ηp^2^, partial eta squared. Bold *p*-values indicate statistically significant main or interaction effects.

**Table 7:** Bonferroni-adjusted *p*-values for bar-position comparisons at each squat depth during the concentric phase.

Muscle	50°	90°	130°
RA	0.116	0.356	0.050
RF	0.761	**0.011**	0.154
VM	1.000	0.077	0.178
VL	1.000	**0.007**	0.210
BF	**0.009**	**0.028**	0.094
ST	**<0.001**	**0.013**	1.000
GM	0.585	0.284	1.000
ES	**0.044**	0.068	0.309

Note. Values are Bonferroni-adjusted *p*-values. RA, rectus abdominis; RF, rectus femoris; VM, vastus medialis; VL, vastus lateralis; BF, biceps femoris; ST, semitendinosus; GM, gluteus maximus; ES, erector spinae. Bold values indicate statistical significance after Bonferroni adjustment.

**Table 8:** Bonferroni-adjusted *p*-values for depth comparisons within each bar position during the concentric phase.

Muscle	HBBS 50–90°	HBBS 50–130°	HBBS 90–130°	LBBS 50–90°	LBBS 50–130°	LBBS 90–130°
RA	**0.041**	**0.016**	**0.041**	1.000	**0.027**	**0.020**
RF	**<0.001**	**<0.001**	**0.003**	**<0.001**	**<0.001**	**<0.001**
VM	**0.005**	**0.001**	**0.006**	**<0.001**	**<0.001**	**0.003**
VL	**<0.001**	**<0.001**	**0.003**	**0.017**	**<0.001**	**0.003**
BF	0.067	**<0.001**	**<0.001**	0.251	**0.002**	**0.004**
ST	0.994	0.308	1.000	1.000	0.435	0.119
GM	0.862	0.371	0.415	0.168	1.000	1.000
ES	**0.002**	**<0.001**	**<0.001**	**0.009**	**<0.001**	**0.003**

Note. Values are Bonferroni-adjusted *p*-values. RA, rectus abdominis; RF, rectus femoris; VM, vastus medialis; VL, vastus lateralis; BF, biceps femoris; ST, semitendinosus; GM, gluteus maximus; ES, erector spinae; HBBS, high-bar back squat; LBBS, low-bar back squat. Bold values indicate statistical significance after Bonferroni adjustment.

## Data Availability

The data presented in this study are available on request from the corresponding author.
